# Uncovering the spectrum of adult zebrafish neural stem cell cycle regulators

**DOI:** 10.3389/fcell.2022.941893

**Published:** 2022-06-29

**Authors:** Aurélien Caron, Lidia Trzuskot, Benjamin W. Lindsey

**Affiliations:** Laboratory of Neural Stem Cell Plasticity and Regeneration, Department of Human Anatomy and Cell Science, Rady Faculty of Health Sciences, University of Manitoba, Winnipeg, MB, Canada

**Keywords:** adult neural stem and progenitor cells, zebrafish, cell proliferation, cell cycle regulation, environmental enrichment, social behaviour, central nervous system repair, neurogenesis

## Abstract

Adult neural stem and progenitor cells (aNSPCs) persist lifelong in teleost models in diverse stem cell niches of the brain and spinal cord. Fish maintain developmental stem cell populations throughout life, including both neuro-epithelial cells (NECs) and radial-glial cells (RGCs). Within stem cell domains of the brain, RGCs persist in a cycling or quiescent state, whereas NECs continuously divide. Heterogeneous populations of RGCs also sit adjacent the central canal of the spinal cord, showing infrequent proliferative activity under homeostasis. With the rise of the zebrafish (*Danio rerio*) model to study adult neurogenesis and neuroregeneration in the central nervous system (CNS), it has become evident that aNSPC proliferation is regulated by a wealth of stimuli that may be coupled with biological function. Growing evidence suggests that aNSPCs are sensitive to environmental cues, social interactions, nutrient availability, and neurotrauma for example, and that distinct stem and progenitor cell populations alter their cell cycle activity accordingly. Such stimuli appear to act as triggers to either turn on normally dormant aNSPCs or modulate constitutive rates of niche-specific cell cycle behaviour. Defining the various forms of stimuli that influence RGC and NEC proliferation, and identifying the molecular regulators responsible, will strengthen our understanding of the connection between aNSPC activity and their biological significance. In this review, we aim to bring together the current state of knowledge on aNSPCs from studies investigating the zebrafish CNS, while highlighting emerging cell cycle regulators and outstanding questions that will help to advance this fascinating field of stem cell biology.

## Introduction

Teleost fishes serve as exceptional models to study the cell cycle dynamics and function of adult neural stem and progenitor cells (aNSPCs) throughout the central nervous system (CNS). The lifelong presence of proliferating aNSPCs across diverse stem cell niches of the brain (Zupanc et al., 2005; Grandel et al., 2006), along with their remarkable neuroregenerative capacity following brain and spinal cord injury (Zupanc and Sirbulescu, 2012) make teleosts extremely attractive to study. These attributes have allowed researchers to take advantage of fish models to study the biological significance of adult neurogenesis (Lindsey and Tropepe, 2006) as well as the process of timely brain and spinal cord repair (Becker and Becker, 2008). Adult neurogenesis is defined as a lineage-directed process that commences with dividing aNSPCs that generate daughter cells fated towards a neuronal phenotype. A major difference between constitutive adult neurogenesis and regenerative neurogenesis that occurs after CNS damage, is that the latter largely relies on the activation of normally quiescent aNSPCs to re-enter the cell cycle. A fundamental question under homeostasis and following injury is what suite of factors are responsible for controlling aNSPC activity.

**FIGURE 1:**
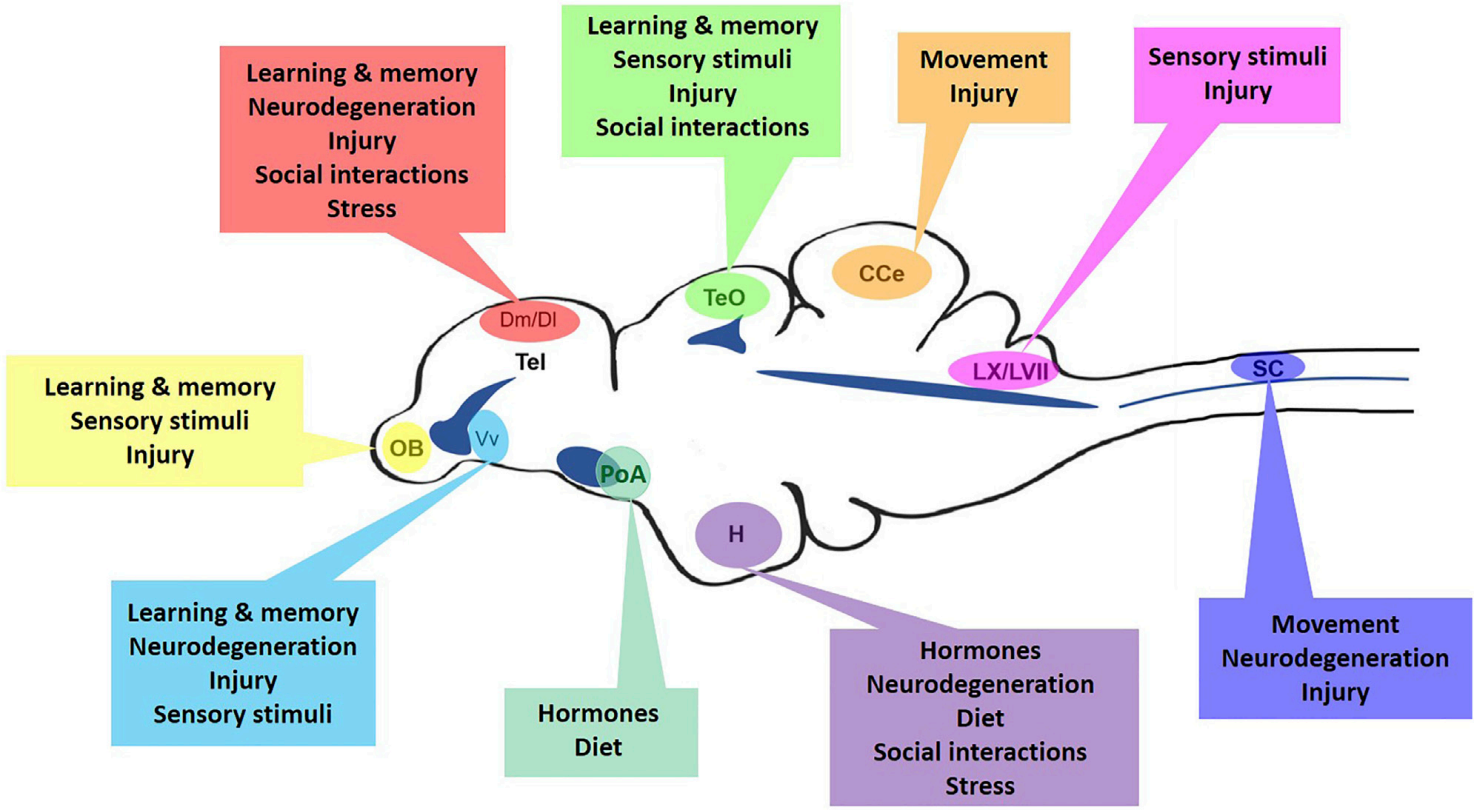
The many regulators of niche-specific aNSPCs in the adult zebrafish CNS. Mid-sagittal view of the zebrafish brain and spinal cord displaying major aNSPC niches and the array of homeostatic, injury-induced, or neurodegenerative regulators that may control their cell cycle dynamics. OB, olfactory bulbs; Tel, telencephalon; Dm, medial dorsal pallium; Dl, lateral dorsal pallium; Vv, ventral ventricular zone; TeO, optic tectum; PoA, preoptic area; H, hypothalamus; CCe, corpus cerebelli; LX/LVII, vagal and facial lobes; SC, spinal cord. Dark blue indicates approximate location of brain ventricles and the spinal cord central canal.

Distinguished as the most diverse vertebrate class and having adapted to nearly every aquatic environment (Kotrschal and Palzenberger, 1992), teleosts are excellent examples to explore how differences in habitat, environment, social interactions, and neurotrauma can impinge upon aNSPC function. The zebrafish (*Danio rerio*) has become one of the most popular laboratory models to study these factors in the brain and spinal cord. The social nature and complex behaviours displayed by this species (Kalueff et al., 2013), in combination with the multitude of molecular tools and small size of the CNS for imaging, has set the zebrafish apart to study neurogenesis and neurorepair. Unlike amniotes (Kriegstein and Alvarez-Buylla, 2009), fish retain developmental stem cell populations over ontogeny, including radial-glial cells (RGCs; aka ependymoglia in the spinal cord) in a quiescent or cycling state, and constitutively proliferating neuro-epithelial cells (NECs). These cells are distributed across diverse integrative and sensory niches of the brain and spinal cord (Lindsey et al., 2018). Therefore, this model offers the opportunity to uncover how unique biological contexts can stimulate aNSPC phenotypes (Figure 1), and help reveal the mechanisms regulating cell cycle activity.

Here, we provide an overview of the recent factors understood to regulate aNSPC cell cycle dynamics in teleosts, focusing on studies from the zebrafish model (summarized in Table 1). A key element of this review is to synthesize our knowledge of how day-to-day environmental stimuli can modulate constitutive rates of cell proliferation; an area poorly investigated in fish. We conclude by discussing key outstanding questions and available techniques to move these forward, to yield novel insight regarding the activity of aNSPCs under physiological and pathological conditions.

**TABLE 1:** Factors impacting aNSPC cell cycle activity in the zebrafish CNS.

Regulator	Stem cell niche	aNSPC	Δ cell cycle	References
Telencephalon				
^a^Estrogen	Whole brain	RGC & NEC	Decrease	Makantasi and Dermon, (2014)
Tank enrichment	Whole Tel	RGC & NEC	Increase	von Krogh et al. (2010)
Male social subordination	DP	RGC	Decrease	Tea et al. (2019)
Social stimulation	DP, PoA	RGC	Increase	Dunlap et al. (2021)
GnRH	PoA	RGC & NEC	Increase	Ceriani and Whitlock, (2021)
Dorsal stab lesion	Tel	RGC	Increase	Kroehne et al. (2011); Kishimoto et al. (2012); Barbosa et al. (2015)
*cxcr5* knock-down	DP (injury)	RGC	Decrease	Kizil et al. (2012a)
Gata3 knock-down	DP (injury)	RGC	Decrease	Kizil et al. (2012b)
BMP/Id1	DP (homeostasis and injury)	RGC	Maintain quiescence	Rodriguez Viales et al. (2015), Zhang et al., 2021
*Mdka*	DP (homeostasis and injury)	RGC	Quiescence	Lübke et al. (2022)
*Notch*	DP (homeostasis and injury)	RGC	Quiescence	Chapouton et al. (2010); Anand and Mondal, (2017)
Inflammation	DP (homeostasis and injury)	RGC	Increase	Kyritsis et al. (2012)
Chronic hyperglycemia	Tel (homeostasis and injury)	RGC	Decrease	Dorsemans et al. (2017), Ghaddar et al. (2021)
Amyloid-β-42	Tel	RGC & NEC	Increase	Cosacak et al. (2019); Bhattarai et al. (2020)
Serotonin	Tel (AD model)	RGC	Decrease	Bhattarai et al. (2020)
BDNF	Tel (AD model)	RGC	Increase	Bhattarai et al. (2020)
IL-4/STAT6	Tel (AD model)	RGC	Increase	Bhattarai et al. (2020)
Serotonin promotion	PoA	RGC	Increase	Thompson et al. (2017)
Midbrain				
Visual deprivation	TeO	NEC	Decrease	Lindsey et al. (2014); Boulanger-Weill et al. (2017); Hall and Tropepe, (2018a)
Serotonin inhibition	H	RGC	Decrease	Pérez et al. (2013)
Serotonin promotion	H	RGC	Increase	Thompson et al. (2017)
Injury	TeO	RGC	Increase	Shimizu et al. (2018); Lindsey et al. (2019)
IL6/Stat3	TeO (injury)	RGC	Increase	Shimizu et al. (2021)
Injury	TeO	NEC	Increase	Shimizu et al. (2018); Lindsey et al. (2019)
Chronic hyperglycemia	MB	RGC & NEC	Decrease	Dorsemans et al. (2017)
Chronic starvation	TeO	NEC	Decrease	Benítez-Santana et al. (2016)
Corpus Cerebelli and Spinal Cord				
Injury	CCe	RGC & NEC	Increase	Kaslin et al. (2017)
Chronic hyperglycemia	CCe	RGC & NEC	Decrease	Stankiewicz et al. (2017)
Exercise	SC	RGC	Increase	Chang et al. (2021)
Injury	SC	RGC	Increase	Reimer et al. (2009)

a Brain-wide stem cell niches affected.

Only studies showing a significant effect on aNSPC proliferation are listed. Tel, Telencephalon; DP, dorsal pallium; PoA, preoptic area; AD, Alzheimer’s Disease; TeO, optic tectum; H, hypothalamus; MB, midbrain; CCe, corpus cerebelli; SC, spinal cord; RGC, radial-glial cells; NEC, neuro-epithelial cells.

## The effect of stress and social behaviour on adult neural stem and progenitor cell activity

Zebrafish are highly social species in the wild and under laboratory conditions (Suriyampola et al., 2016). Social interactions can take the form of predator-prey encounters, mating opportunities, conspecific relationships, and the formation of social hierarchies. This daily social plasticity commonly involves one or more chemosensory or visual cues, as well as the possibility of changes in swimming performance. This suggests that a broad range of adult niches could be implicated in aNSPC dynamics, including those processing sensory input and potentially even the spinal cord where additional motor neurons may be required to accommodate increased swimming. A consequence of these interactions is their effect on the physiology of the animal, such as the stress axis, and how this information acts to control aNSPCs behaviour.

In thinking how social behaviours modulate aNSPC activity, a key question is what constitutes a physiological stressful event. Zebrafish RGCs are known to possess the glucocorticoid receptor Nr3c1 (Nelson et al., 2019), making them sensitive to changes in circulating cortisol. Higher cortisol levels have been identified in group-housed compared to individually-housed zebrafish (Onarheim et al., 2022), arguing against the idea that isolation consistently increases stress in social animals. One hypothesis may be that group composition, including sex ratios, fish size, and potential for hierarchies, are potential drivers of stress levels. Cues in the environment of isolated fish further appear to play a role in regulating cortisol levels. This has been illustrated by zebrafish having higher cortisol in an enriched-isolated context compared to a barren-isolated context (von Krogh et al., 2010). Work by Lindsey and Tropepe (2014) rather found that cortisol levels strongly correlated with social context, with the effect on aNSPC proliferation or neuronal differentiation being niche-specific. Social novelty and isolation revealed lower cortisol levels than grouped animals, with aNSPCs in sensory niches having the largest reduction in cell cycle activity. This work highlights the importance of pre-existing social experiences in shaping the future stress response and probability of stimulating niche-specific aNSPCs.

A small number of studies have also centered around the role of social hierarchies in driving aNSPC behaviour. One interesting report focused on sex-specific differences. In this study, the authors illustrated that subordinate males displayed reduced cell proliferation and neurogenesis in the dorsal telencephalon, along with increased cortisol levels (Tea et al., 2019). In females, however, no change was observed in dominant or subordinate animals when compared to group-housed controls. This suggests the possibility that hormonal differences may play a part in the cellular activity of aNSPCs. This finding aligns with earlier work studying socially suppressed subordinate male cichlids, where cell proliferation in the brain was lowest compared with dominant animals (Maruska et al., 2012). Notably, this study showed that if males were given the opportunity to rise in rank, the proportion of dividing aNSPCs increased in parallel. In the zebrafish, we now understand that changes in social status are closely correlated with gene expression patterns involved in neural plasticity in a niche-specific manner (Teles et al., 2016).

## The emerging role of hormones and diet on adult neural stem and progenitor cell regulation

In the past few years, newer studies have emerged addressing the role of hormones and diet in regulating aNSPC activity in the zebrafish. This recent focus could provide valuable insight towards the importance of sex-specific differences, reproductive cycles, seasonality, food availability and nutrient composition in teleost fish. Several of these factors could also be increasingly important to consider during the design phase of experiments aimed at studying adult neurogenic plasticity.

Interestingly, in zebrafish only RGCs and not NECs are known to express the estrogen-synthesizing enzyme, aromatase-B (Pellegrini et al., 2016). Upon administration of estrogen to female zebrafish a reduction in cell proliferation in multiple brain regions has been reported, with the greatest impact in the dorsal/ventral telencephalon, preoptic area, hypothalamus, and cerebellum (Makantasi and Dermon, 2014). Within the niche of the preoptic area, animals treated with gonadotropin releasing hormone, but not testosterone produce an increase in cell division in non-RGC stem cell populations (Ceriani and Whitlock, 2021). Alternatively, inhibiting aNSPCs residing in the hypothalamus using the serotonin blocker tryptophan hydroxylase attenuates aNSPC proliferation, illustrating the dependency of stem cells in this domain on serotonin (Pérez et al., 2013).

How zebrafish diet or feeding regime relates to proliferative behaviour of aNSPCs is poorly characterized to date. Currently, studies have taken the form of either exposure to a high fat diet or starvation. Chronic hyperglycemia in adult zebrafish, for instance, was reported to impair homeostatic neurogenesis in the telencephalon, midbrain, and cerebellum, while also having a pro-inflammatory and oxidative stress effect (Dorsemans et al., 2017; Stankiewicz et al., 2017; Ghaddar et al., 2021). Conversely, 10 weeks of reduced food intake appears to be insufficient to alter aNSPC activity in the forebrain (Arslan-Ergul et al., 2016), but a 5 week starvation is adequate to decrease aNSPC proliferation in the optic tectum (Benítez-Santana et al., 2016). Additional niche-wide studies focusing on food type, nutrient composition, and feeding frequency undoubtedly would be beneficial to determine if distinct aNSPC populations are differentially affected and how this relates to changes in cell metabolism.

## Sensory stimuli as a potent driver of adult neural stem and progenitor cell activity

Sensory input is a potent regulator of animal behaviour. In an aquatic environment teleosts receive this information from visual cues, olfactory and taste cues (i.e., chemosensory), as well as lateral line input (i.e., mechanosensory). A unique feature of zebrafish is that aNSPCs exist in neurogenic niches within primary processing sensory structures of the mature brain, including the forebrain olfactory bulbs (smell), midbrain optic tectum (vision), and hindbrain vagal/facial lobes (taste; Zupanc et al., 2005). These sensory domains offer the ability to study a range of modality-specific cues and their effect on aNSPC lineage activity. With several teleost models having conserved sensory niches, including the zebrafish, medaka, brown ghost knifefish, goldfish, and killifish (Ganz and Brand, 2016), this field is wide open to compare the functional role of aNSPCs using species-specific biologically relevant forms of sensory stimuli.

Studies applying forms of environmental enrichment or selective visual cues have shown a strong link between sensory input and aNSPC activity. A pointed example of how the mere opportunity for visual stimuli can induce changes in long-term aNSPC activity comes from recent work by Dunlap et al. (2021). Here, socially acclimated zebrafish that were first isolated, before being exposed to conspecifics in an adjacent tank chamber, was sufficient alone to enhance forebrain aNSPC activity. The above finding is supported by an early study exposing zebrafish to an enriched environment adorned with aquatic plants and gravel, or devoid of such items, showing a general increase in proliferation of forebrain aNSPCs with enrichment (von Krogh et al., 2010). An outstanding question is what effect does visual enrichment have on parent or progenitor NECs along the tectal marginal zone (Lindsey et al., 2018a), that would be predicted to be modulated.

Visual and chemosensory assays have also been employed to more directly test the effect of modality-specific sensory input on the activity of aNSPCs in corresponding sensory niches. Exposing zebrafish to monochromatic light has been demonstrated to decrease the proportion of cycling aNSPCs in the tectal sensory niche (Lindsey et al., 2014). Experiments using larval zebrafish also show that visual restriction impedes neurogenesis and functional integration into the optic tectum, correlating with reduced BDNF production (Boulanger-Weill et al., 2017; Hall and Tropepe, 2018a). In contrast, a 7-day treatment using a chemosensory assay resulted in an increase in neuronal survival but limited effect on RGC proliferation in the bulbs and vagal lobe (Lindsey et al., 2014). A major finding from this work was that modality-specific sensory input triggered the relevant sensory processing niche, and not those coding alternative modalities.

Taking advantage of sensory niches in the adult zebrafish brain further offers the chance to study the functional significance of aNSPCs for learning. Using sensory paradigms to explore the impact of task complexity on endogenous neurogenesis arising from sensory zones would provide new insight not possible in mammals. For instance, building on previous olfactory learning paradigms (Braubach et al., 2009), researchers can now ask how olfactory learning might modulate aNSPC activity along the Rostral Migratory Stream (RMS; Adolf et al., 2006; Kishimoto et al., 2012) as compared to resident RGC behaviour directly in the bulbs. This would provide valuable comparative data with mammals who maintain a lifelong RMS (Bond et al., 2015). In addition, since learning implicitly involved a memory component, with the dorsal lateral niche of the telencephalon homologous to the mammalian hippocampus (Ganz et al., 2015), the combined activity of aNSPCs in sensory and cognitive niches can be examined.

Modulation of spinal cord RGCs for the most part has not been explored as this population resides mainly in a quiescent state. Recently, one of the first studies of its kind has shown that zebrafish subjected to increased swimming using a swim tunnel was sufficient to enhance cell cycle proliferation of normally dormant spinal cord ependymoglia, and the generation of newborn motor neurons (Chang et al., 2021). Applying the same paradigm after spinal cord injury showed a similar trend, proposing that increased swimming exercise modulates neurogenesis. Conversely, experiments performing movement restraint in larval zebrafish have illustrated a decrease in neural stem and progenitor proliferation in the developing forebrain, though how robust this effect is across the neuro-axis remains unknown (Hall and Tropepe, 2018b). Together, these studies provide early support for the role of “exercise” in modulating baseline levels of aNSPCs in the brain and spinal cord of teleost models.

## Cell intrinsic and injury-induced signals regulate adult neural stem and progenitor cells in the damaged central nervous system

Much effort has been placed on understanding the behaviour of RGCs and NECs following CNS damage compared to homeostatic modulation. More recently, this has also included examination of aNSPCs in a neurodegenerative context. Common to brain and spinal cord injury is the involvement of quiescent RGCs that are awakened following insult to re-enter the cell cycle and repopulate lost neuronal subtypes. In some instances, constitutively active NECs have been shown to additionally contribute to repair. Growing evidence suggests that aNSPCs retain diverse regenerative capacities that differ by stem cell niche in the adult CNS (Lindsey et al., 2018a), but their role in neurodegenerative diseases is only now beginning to be uncovered. This implies the need to better understand the balance between the factors comprising the damaged stem cell niche, and the intrinsic regenerative programs of distinct aNSPC populations. With many excellent reviews already published on this topic (Alunni and Bally-Cuif, 2016; Alunni et al., 2020), here we strive to provide a brief synopsis of the newest developments in this area to contrast with the regulators of aNSPCs observed under physiological conditions.

In the forebrain and spinal cord, RGCs drive the repair process. Accordingly, dorsal forebrain stab injury leads to the activation of RGCs, in addition to oligodendrocyte precursor cells (OPCs), that serve to replenish lost neurons and oligodendrocytes, respectively (Kroehne et al., 2011; Baumgart et al., 2012; Kishimoto et al., 2012; Barbosa et al., 2015; Sanchez-Gonzales et al., 2022). Most recently, transcriptomic analysis has provided insight regarding early proliferative signatures of RGCs and OPCs in the telencephalon and spinal cord after injury (Tsata et al., 2020; Demirci et al., 2022; Sanchez-Gonzales et al., 2022), adding to our existing knowledge of the role of *cxcr5* and Gata3 during neurorepair (Kizil et al., 2012a; 2012b). In the dorsal forebrain, genes regulating stem cell quiescence under homeostasis, such as *mdka*, have also recently been shown to continue to be expressed following brain injury in non-reactive, RGC populations, suggesting a potential mechanism to prevent NSPC pool exhaustion (Lübke et al., 2022). This finding will add valuable information to our deep-rooted understanding of the role of Notch signalling in maintaining the quiescent state of NSPCs (Chapouton et al., 2010; Anand and Mondal, 2017). In the spinal cord, upon injury ependymoglia enter the cell cycle to produce motor neurons for functional swimming recovery (Reimer et al., 2009). In both the forebrain and spinal cord, numerous developmental regulatory pathways, such as Wnt, Notch, Shh, and ID1/BMP, are recapitulated following injury (Cardozo et al., 2017; Alunni et al., 2020; Zhang et al., 2021). Additionally, studies in the adult forebrain (Kyritsis et al., 2012) and larval spinal cord (Tsarouchas et al., 2018) confirm that activation of the immune response post-injury is critical to induce RGCs to regenerate. The rapid repair offered by the larval zebrafish following spinal cord injury has also gained traction as an efficient model for drug screening to identify therapeutics to test in mammals to improve spinal cord repair (Chapela et al., 2019; Nelson and Granato, 2022). Such studies equally afford the opportunity to examine how NSPC proliferative activity and intrinsic signaling pathways are modulated with application of these small molecules in an organism capable of successful spinal cord regeneration. Comparing the interaction between the immune response and RGCs or OPCs of the mature brain and spinal cord would be of great interest to identify CNS-wide themes in neuroregeneration. Unfortunately, detailed studies investigating the immune response following spinal cord injury in juvenile and adult stages are still lacking.

A small number of studies have also underscored the contribution of constitutively cycling NECs following brain injury. In the optic tectum, quiescent RGCs reside along the roof of the tectal ventricle whereas a pool of NECs sit at the midline tectal marginal zone, continuously furnishing the optic tectum with a small number of newborn neurons (Ito et al., 2010). Following tectal injury, it was reported that NECs amplify their cell cycle rates and neuronal output, while progeny of activated RGCs produce only newborn RGCs (Lindsey et al., 2019). Tectal injuries more proximal to the NEC population and in younger adult animals nevertheless showed evidence that RGCs could generate newborn neurons (Shimizu et al., 2018), suggesting that adult age appears to play a role in the regenerative potential of aNSPCs (Edelmann et al., 2013). This and more recent work has implicated Wnt and IL-6/STAT3 pathways in regulating RGC activity after injury in the tectum (Shimizu et al., 2018; 2021). In the adult cerebellum, NECs also appear to be the main contributor to restorative neurogenesis (Kaslin et al., 2017). However, in juvenile fish, RGCs of the cerebellum play a more prominent role in the regenerative process alongside NECs, giving rise to neuronal phenotypes similar to constitutive neurogenesis. Collectively, work on RGCs and NECs provide growing evidence that the regenerative process in zebrafish is accomplished by a combination of reactivated RGCs and constitutively proliferating NECs that are influenced by cellular senescence.

Models of Alzheimer’s and Parkinson’s disease have also been established in the zebrafish, permitting studies of aNSPC activity during the process of neurodegeneration. Alzheimer’s models along with single-cell transcriptomics have illustrated neurodegenerative specific regulation of aNSPCs. These studies show that induced inflammation leads to a cascade of events, initiated by the activation of the IL4/STAT6 pathway. Subsequent downregulation of serotonin metabolism and promotion of BDNF in turn increase aNSPCs proliferation in a subtype and niche-specific manner (Bhattarai et al., 2016; Cosacak et al., 2019; Bhattarai et al., 2020). Parallel studies in older adult zebrafish showed similar levels of aNSPC proliferation, but a diminished immune response and fewer newborn neurons (Bhattarai et al., 2017). Modelling Parkinson’s disease in the zebrafish, reports have shown that ablation of dopaminergic neurons leads to inflammation and RGC progenitor activity in the diencephalon to repopulate newborn dopaminergic neurons (Caldwell et al., 2019). Inducible transgenic lines have also come available to temporally ablate dopaminergic neurons (Godoy et al., 2015), providing a valuable tool to better interrogate this disease. While still new to the field of neurodegeneration, the zebrafish model offers many excellent advantages to elucidate the response of aNSPCs following Alzheimer’s or Parkinson’s disease that have yet to be capitalized upon.

## Outstanding questions and future directions

The zebrafish is a tractable model to probe the mechanisms regulating aNSPC function. Still many outstanding questions persist. First, many niches of the brain are composed of populations of both quiescent and actively cycling RGCs. Adult neurogenesis by definition focuses on constitutively proliferating cells, whereas CNS regenerative studies concentrate primarily on the reactivation of quiescent aNSPCs. Whether certain forms of stimuli under homeostasis are sufficient to push quiescent RGCs into a cycling state, or a subpopulation of normally dividing RGCs of the forebrain might contribute to regeneration, is unclear. Equally unknown is whether RGCs or NECs can undergo shifts in their multipotency to generate specific neuronal lineages under different contexts, and if so, what are the intrinsic programs or changes in the microenvironment responsible for this cellular plasticity? Second, without knowledge of the molecular signature of similar aNSPCs found in different adult niches, it is unclear whether their gene expression profiles align with their varied response to environmental or injury-induced cues. Many cell autonomous features also remain to be understood, including cell metabolism (Santoro, 2014), senescence (Da Silva-Álvarez et al., 2020), and the role of the unfolded protein response (Clark et al., 2020). Considering the stem cell niche, most of our knowledge is limited to the role of the immune response post-injury in activating aNSPC populations. Studying the involvement of neighboring cells, the extracellular matrix (Kim et al., 2018), and vasculature (Chen et al., 2019) is paramount to gain broader knowledge of niche-specific regulation of aNSPCs for successful neurorepair.

Methods to study the activity of zebrafish aNSPCs are extensive. These include the use of thymidine analogues such as BrdU and EdU, that label cells undergoing DNA synthesis (Cavanagh et al., 2011), as well as the endogenous cell cycle marker PCNA (Schönenberger et al., 2015). The dual Fucci line further allows changes in cell cycle phases to be fluorescently monitored (Bouldin and Kimelman, 2014); a generally underutilized tool thus far. More recent tamoxifen-inducible Cre-lox systems in zebrafish have allowed temporal colour-switching of aNSPCs to monitor population dynamics (Mosimann et al., 2011). Above all, the zebrabow system permits the most advanced multi-colour labeling to faithfully track stem cell lineages arising from parent stem cells (Pan et al., 2013). This suite of tools provides the opportunity to gain deeper insight towards aNSPC context-dependent activity. Combining these techniques with the multiple imaging approaches in the zebrafish, including live *in vivo* studies, 3-dimensional imaging of the CNS (Lindsey et al., 2018b), and advanced electron microscopy (Oorschot et al., 2021), the next decade promises to hold many exciting discoveries regarding teleost aNSPC activity, regulation, and biological significance.
